# GABA-Producing *Lactococcus lactis* Strains Isolated from Camel’s Milk as Starters for the Production of GABA-Enriched Cheese

**DOI:** 10.3390/foods10030633

**Published:** 2021-03-17

**Authors:** Begoña Redruello, Yasmine Saidi, Lorena Sampedro, Victor Ladero, Beatriz del Rio, Miguel A. Alvarez

**Affiliations:** 1 Dairy Research Institute (IPLA-CSIC), Paseo Rio Linares s/n, 33300 Villaviciosa, Spain; bredruel@ipla.csic.es (B.R.); loresampedro07@gmail.com (L.S.); ladero@ipla.csic.es (V.L.); maag@ipla.csic.es (M.A.A.); 2 Applied Microbiology Laboratory, Department of Biology, Faculty of Nature and Life Sciences, University of Oran, Oran 31000, Algeria; yasmine.saidi@gmail.com; 3 Instituto de Investigación Sanitaria del Principado de Asturias (ISPA), Hospital Universitario Central de Asturias, 33006 Oviedo, Spain

**Keywords:** camel’s milk, *Lactococcus lactis*, GABA, starter cultures, functional cheese

## Abstract

The multiple health benefits attributed to the bioactive compound γ-aminobutyric acid (GABA) have prompted the food industry to investigate the development of functional GABA-rich foods via the use of GABA-producing microorganisms. This study reports the isolation of six GABA-producing *Lactococcus lactis* strains from camel’s milk; this is the first time that such microorganisms have been isolated from milk. The sequencing and *in silico* analysis of their genomes, and the characterisation of their technological and safety properties, confirmed their potential as starters. Experimental cheeses made with all six strains (individually) accumulated GABA at concentrations of up to 457 mg/kg. These GABA-producing *L. lactis* strains could be used as starter cultures for the manufacture of functional GABA-enriched cheeses that provide health benefits to consumers.

## 1. Introduction

*Lactococcus lactis* is the lactic acid bacterium (LAB) most widely used as a primary fermentation starter in the dairy industry. It is routinely employed in the production of matured cheese, unripened cheeses (e.g., cream cheeses and cottage cheese), fermented milk products, sour cream, and fermented butter. Based on its traditional usage in the fermentation of food, this species has been granted Qualified Presumption of Safety (QPS) status by the European Food Safety Authority (EFSA, [1]) and it enjoys Generally Regarded as Safe (GRAS) status in the United States [2]. *L. lactis* plays a major role during the early stages of cheesemaking, since it quickly acidifies milk by metabolizing the lactose present into lactic acid [3]. This lactic acid inhibits the growth of spoilage and pathogenic microorganisms, improving the shelf life and safety of the fermented end product. Some *L. lactis* strains also improve product preservation via the release of antimicrobial substances such as organic acids, H_2_O_2_, and bacteriocins, which inhibit the growth of undesirable (e.g., *Clostridium* and *Bacillus*) and pathogenic Gram-positive bacteria (e.g., *Staphylococcus aureus* and *Listeria monocytogenes*) [4]. The species also plays a crucial role during cheese ripening via its proteolytic action, which contributes to the formation of compounds involved in the final product’s organoleptic properties (flavour, taste, and texture) [3].

Some strains of *L. lactis* also show interesting probiotic potential. Several studies have shown that certain strains of *L. lactis* are beneficial to human health via their anti-inflammatory [5], immunomodulatory [6], and antioxidant [7] activities. Some strains are known to produce γ-aminobutyric acid (GABA), which can protect against the neurodegeneration induced by injury and help prevent neurological disorders [8]. It also has blood pressure-lowering and anti-diabetic properties, and it exerts an anti-cancer effect through the induction of apoptosis and the inhibition of cancer cell proliferation and the production of metastases. In addition, it is intestine-, hepato-, and cardio-protective, and it has positive effects against anxiety and depression [8]. The fact that GABA has also been approved as a food ingredient in the US and EU have led the pharmaceutical and food industries to show interest in developing functional GABA-based supplements and GABA-enriched foods [8].

No GABA-producing *L. lactis* strains have ever been isolated from milk. However, dairy products such as fermented milk [9], yoghurt [10], and cheese [11] have been made with GABA-producing LAB isolated from other sources. These products are claimed to have anti-hypertension [9,12] and anti-diabetic effects [10]. GABA-enriched cheeses have been made using adjunct cultures of different GABA-producing LAB species, such as *Levilactobacillus brevis* [13], *Lacticaseibacillus casei* [14], *Lacticaseibacillus paracasei* [15], *Lactiplantibacillus plantarum* [15], *Lentilactobacillus buchneri* [16], *Streptococcus thermophilus* [13], and *L. lactis* [12,17,18].

GABA is produced by *L. lactis* via the enzymatic decarboxylation of glutamic acid. The genes involved in this reaction are grouped into the GAD cluster, the transcriptional organization of which was first described in *L. lactis* MG1363 by Sanders et al. [19]. The GAD cluster of *L. lactis* consists of three genes: *gadR*, which codes for a positive transcriptional regulator (GadR), *gadC*, which lies downstream and codes for the glutamate–GABA antiporter (GadC), and finally *gadB*, which codes for glutamate decarboxylase (GadB; the enzyme that catalyzes the decarboxylation of glutamate to GABA). The GAD pathway contributes to the acid resistance shown by *L. lactis* in acidic environments [19], which is a property seen in other LAB such as *Limosilactobacillus reuteri* [20] and indeed other types of bacteria such as *Escherichia coli* and *Listeria monocytogenes* [21].

The concentration of GABA in camel’s milk is high, certainly far more so than in cow’s and human milk [22], suggesting it could be a source of GABA-producing *L. lactis* strains that might have applications in the dairy industry. The objectives of the present work were to isolate GABA-producing *L. lactis* strains from raw camel’s milk and to perform the characterization of their genomic, technological, and safety properties. Finally, the potential of these strains as starter cultures for the production of functional GABA-enriched cheeses was assessed.

## 2. Materials and Methods 

### 2.1. Bacterial Strains and Growth Conditions

Table 1 lists the bacterial strains used in this study. *L. lactis* cultures were grown in De Man, Rogosa and Sharpe (MRS) or M17 broth (Oxoid, Basingstoke, UK) supplemented with 0.5% (*w*/*v*) glucose (GM17) at 30 °C without aeration. *Streptococcus thermophilus* strains were grown in M17 broth supplemented with 0.5% (*w*/*v*) lactose and incubated at 42 °C without aeration. *Latilacctobacillus sakei* was grown in MRS at 30 °C without aeration. *Listeria innocua* was grown in Tryptic Soy Broth (TSB) broth (Oxoid) at 37 °C without aeration. *Micrococcus luteus* was grown in TSB broth at 37 °C with shaking.

### 2.2. Milk Sample Collection

Twelve milk samples were obtained directly from the udder of lactating camels that came from eight different areas of Algeria (Abadla, Adrar, Bechar, Ghardaia, Mecheria, Oran, Saida, and Tindouf) between October 2015 and October 2017. Samples were kept at 4 °C until processed. 

### 2.3. Isolation of Lactic Acid Bacteria and Phenotypic Characterization 

To first isolate LAB species, serial dilutions of camel’s milk were plated in duplicate on GM17 agar and incubated at 30 °C. Then, those isolates were subjected to Gram staining and catalase activity [26] and spore formation analyses [27]; those that met the appropriate criteria were identified as LAB.

To identify *Lactococcus* species among these LAB, cell morphology was examined by optical microscopy. Isolates were also grown in GM17 broth at 30 °C and 45 °C, in the presence of 4% and 6.5% NaCl, adjusted to pH 9.6, with inverted Durham tubes to determine CO_2_ production from glucose. Those ovococci able to grow at 45 °C, at pH 9.6, and in the presence of 6.5% NaCl were assigned to *Enterococcus*, while those able to resist pH 9.6, and/or a temperature of 45 °C, but not the presence of 6.5% NaCl, were assigned to *Lactococcus* [28].

Then, *Lactococcus* isolates were grown in M16BCP medium (containing 2 mg/mL lactose, 4 mg/mL arginine, with bromocresol purple as a pH indicator) to confirm their capacity to hydrolyze arginine via the action of arginine dihydrolase (ADH) [also known as arginine deiminase (ADI)].

### 2.4. Molecular Identification of Lactococcus Isolates

The *Lactococcus* isolates were identified at the species level by *16S rRNA* gene sequencing following the protocol described in Saidi et al. [29]. 

### 2.5. Identification of GABA-Producing Lactococcus in Culture Media 

GABA production was detected in supernatants obtained after 5 days of incubation in 1 mL of GM17 broth supplemented with 5 mM L-glutamic acid monosodium salt monohydrate (monosodium glutamate) (Sigma-Aldrich, Madrid, Spain). Culture supernatants were derivatized with diethyl ethoxymethylenemalonate (Sigma-Aldrich, Munich, Germany) and subjected to ultra-high performance liquid chromatography (UHPLC) using a Waters H-Class ACQUITY UPLC apparatus with a UV detector (Waters, Milford, MA, USA), following the procedures described by Redruello et al. [30].

### 2.6. Whole-Genome Sequencing and Bioinformatic Analyses

Total DNA from *L. lactis* isolates was isolated from 1.5 mL of an overnight culture. Cell pellets were collected by centrifugation and washed with 1 mL of PBS. Then, the cells were resuspended in a lysis buffer containing 20 mg/mL lysozyme (Merck, Madrid, Spain), 200 U mutanolysin (Sigma-Aldrich), 2 mg/mL RNase (AppliChem, Darmstadt, Germany), 20 mM Tris–HCl (pH 8.0), 2 mM EthyleneDiamineTetraAcetic acid (EDTA), and 1.2% Triton X-100 (Merck). Then, this lysis suspension was incubated at 37 °C for 1 h and DNA extracted using the DNeasy Blood and Tissue Kit (Qiagen, Hilden, Germany) according to the manufacturer’s instructions. DNA was stored at 4 °C until analysis. A 0.5 kbp genomic library was constructed and subjected to 150 bp paired-end sequencing (providing approximately 25-fold coverage) using a HiSeq 1000 system sequencer (Illumina, San Diego, CA, USA) (performed at GATC [Eurofins Genomics, Ebersberg bei München, Germany]). Quality filtered reads were assembled using SPAdes software v.3.13.0. Annotation was performed using the Prokaryotic Genomes Annotation Pipeline (PGAP) [31] on the NCBI server, improving the results obtained in BLAST analyses [32]. Gene clusters involved in technologically relevant features were identified by blast comparison. Bacteriocin clusters were analyzed with BAGEL 4 software [33]. The genome sequences of six GABA-producing *L. lactis* strains—here denominated LEY6, LEY7, LEY8, LEY11, LEY12, and LEY13—were deposited with BioProject PRJNA596610 in the NCBI BioProject database (http://www.ncbi.nlm.nih.gov/bioproject/596610, accessed on 27 January 2021) under the accession numbers SAMN13634154, SAMN13634584, SAMN13634590, SAMN13634615, SAMN13634616, and SAMN13634617 respectively. A comparative analysis of the genes present in the genomes of these six strains (core and accessory genome) was performed using Roary v.3.11.2 software [34]. The generated cluster tree was visualized with Phandango v.1.3.0 [35]. 

### 2.7. Acidifying Capacity

Ultra-high temperature (UHT) skimmed cow’s milk were inoculated with overnight cultures of the six GABA-producing *L. lactis* strains (1% (*v*/*v*), 10^7^ cfu/mL) and incubated at 30 °C for 18 h. The pH values were recorded after 6 and 18 h of incubation, and milk clotting was assessed at the end of fermentation. The experiment was performed in triplicate. 

### 2.8. Proteolytic Activity

The proteolytic activity of the GABA-producing *L. lactis* strains was examined by a qualitative method on Plate Count Agar (PCA) (Oxoid) supplemented with 2% UHT skimmed cow’s milk as described by Saidi et al. [29]. In addition, proteolytic activity was quantitatively determined using the O-phthaldialdehyde (OPA) method following the protocol described by the same author. Positive controls involved *L. lactis* strains known for their good proteolytic activity (i.e., *L. lactis* NCDO 604^T^ and *L. lactis* SH4109 [Table 1]). Negative controls (non-inoculated UHT skimmed milk samples incubated under the same conditions) were run in parallel.

### 2.9. Dextran Production

Dextran production was determined using Mayeux, Sandine and Elliker (MSE) agar medium rich in sucrose as described in Saidi et al. [29].

### 2.10. Production of Volatile Compounds

To determine the production of volatile compounds, UHT skimmed cow’s milk was inoculated with individual overnight cultures of the GABA-producing *L. lactis* strains at 1% (*v*/*v*) and incubated for 24 h at 30 °C. A head space gas chromatograph (Agilent Technologies, Wilmington, DE, USA) connected to a mass spectrophotometer detector (HS/GC/MS) was used to quantify volatile compounds [29]. They were quantified as the normalized value of their chromatogram peak area using cyclohexanone (3.6 µg/mL) as an internal standard, which was given a value of 100. Negative controls (non-inoculated UHT skimmed cow’s milk samples) were performed in parallel. 

### 2.11. Production of Antimicrobial Substances

To determine the antimicrobial activity of the isolates, well-diffusion assays were performed according to Saidi et al. [29]. The strains used as microbial indicators are listed in Table 1. The antimicrobial activity of microbially secreted molecules was assessed by measuring the zone of inhibition (including the well diameter) that appeared after 24 h of incubation. 

### 2.12. Production of Biogenic Amines 

The production of the biogenic amines (BA) tyramine, histamine, putrescine, and cadaverine from their respective precursor amino acids (tyrosine, histidine, agmatine, ornithine, and lysine) was assessed as described by Saidi et al. [29] with minor modifications (GM17 broth, instead of MRS, was supplemented with 1 mM of the corresponding amino acid substrate). The accumulation of BA in the supernatant of cell cultures was quantified as described in Section 2.5.

### 2.13. In Silico Prediction of Antimicrobial Resistance Genes

The draft genome sequences of the *L. lactis* strains were screened for the presence of antimicrobial resistance genes using ResFinder v.3.2 software [36] and Resistance Gene Identifier software (RGI v.5.1.0) [37]. The RGI criteria used in the analysis were perfect, strict, complete genes only, and high quality/coverage. 

### 2.14. Experimental Cabrales-Like Mini Cheeses

The six *L. lactis* strains described in Table 1 were used individually as starter strains to produce experimental Cabrales-like mini cheeses. Overnight cultures of *L. lactis* isolates (10^6^ cfu/mL) were individually inoculated into sterilized bottles containing 200 mL of commercial UHT cow’s milk; the mold *Penicillium roqueforti* 1AM8 (10^3^ cfu/mL) and CaCl_2_ (0.02% *w*/*v*) was also added to the bottles. Then, the mixtures were left at 30 °C for 2 h to initiate the growth of the cultures. After this time, liquid rennet extract of bovine and ovine (lamb) origin (Nievi, Vizcaya, Spain) was added as indicated by the manufacturer. The bottles were inverted three times and left to coagulate at 30 °C until the curd acquired the appropriate consistency; then, it was cut, and the bottles were inverted for 20 min to promote draining before centrifugation at 220× *g* for 10 min at room temperature to remove all the whey. All steps were performed under sterile conditions. The mini-cheeses produced were kept in closed, screw-capped sterile jars in a ripening chamber at 15 °C for 90 days. Then, one sample from each mini cheese was obtained, and their GABA, glutamic acid, tyramine, histamine, putrescine, cadaverine, and agmatine contents were determined by UHPLC as described by del Rio et al. [25].

### 2.15. Statistical Analysis

A Student *t*-test was performed for mean comparison between two groups. Mean comparison among three or more groups was conducted via one-way analysis of variance (ANOVA) test followed by a pairwise comparison Tukey post-hoc test. Significance was set at *p* < 0.05. Statistical analysis was carried out using the open source R software (v. 3.5.3) (https://www.r-project.org/, accessed on 27 January 2021).

## 3. Results

### 3.1. Phenotypic Characterization and Molecular Identification of LAB Isolates

Fourteen isolates that were Gram-positive, catalase negative, and non-spore-forming, and thus considered to be LAB, were initially selected. The morphology of their cells, as observed by optical microscopy, indicated 12 isolates (86%) to be ovococci and two (14%) to be bacilli. The ovococci were phenotypically characterized (Table 2). All the isolates were able to hydrolyze arginine; none produced CO_2_ and were therefore considered homofermentative. Six of these ovococci were able to grow at 45 °C, pH 9.6, and in the presence of 6.5% NaCl, suggesting they belonged to the genus *Enterococcus* (data not shown). 

The other six were able to resist pH 9.6, and/or a temperature of 45 °C, but they did not grow in the presence of 6.5% NaCl. These latter strains were assigned to the genus *Lactococcus*, since it has been reported that some *L. lactis* strains isolated from camel’s milk are thermotolerant (resist up to 50 °C) and/or are able to grow at pH 9.6 [28]. *16S rRNA* gene sequencing confirmed that all six to belong to *Lactococcus lactis* subsp. *lactis*; they were named *L. lactis* LEY6, *L. lactis* LEY7, *L. lactis* LEY8, *L. lactis* LEY11, *L. lactis* LEY12, and *L. lactis* LEY13 (Table 1) (hereafter LEY6, LEY7, LEY8, LEY11, LEY12, and LEY13). These strains came from milk samples obtained from different geographical locations; LEY6, LEY7, and LEY8 were in milk from the region of Saida, LEY11 was in milk from Bechar, LEY12 was in milk from Ghardaia, and LEY13 was in milk from Tindouf (Table 2).

### 3.2. All six L. lactis Isolates Produced GABA in Culture Media

**Figure 1:**
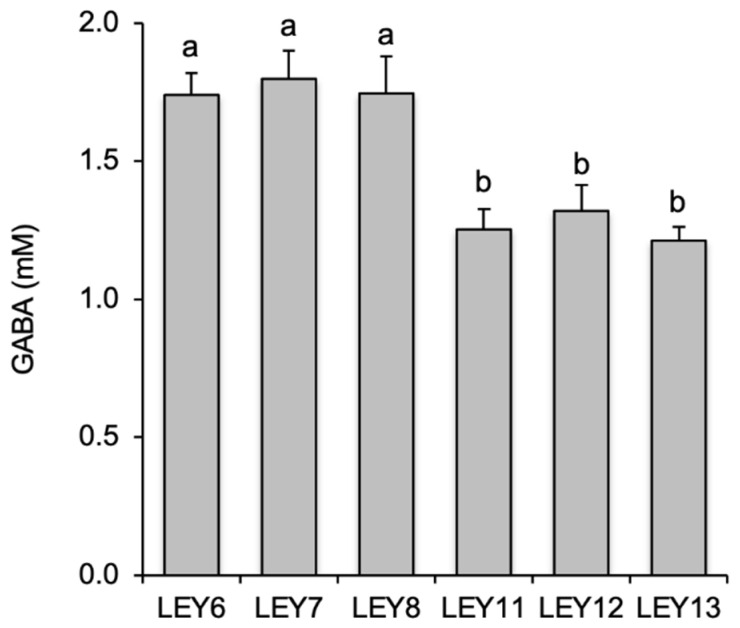
Production of γ-aminobutyric acid (GABA) by *L. lactis* isolates grown in GM17 supplemented with 5 mM of monosodium glutamate for 5 days. Supernatants were analyzed by ultra-high performance liquid chromatography (UHPLC) to determine the GABA concentration of the extracellular medium. Bars with different letters indicate significant differences (*p* < 0.05).

All six selected *L. lactis* isolates produced GABA (Figure 1). LEY6, LEY7, and LEY8 produced significantly greater amounts (from 1.74 ± 0.08 mM for LEY6, to 1.80 ± 0.10 mM for LEY7) than did LEY11, LEY12, and LEY13 (ranging from 1.22 ± 0.05 mM for LEY13, to 1.32 ± 0.09 mM for LEY12). No significant differences were seen between the amount of GABA produced by LEY6, LEY7, and LEY8, nor between LEY11, LEY12, and LEY13.

### 3.3. Whole-Genome Sequencing and Comparison Confirmed the Six GABA-Producing L. lactis to Be Different Strains

**Figure 2:**
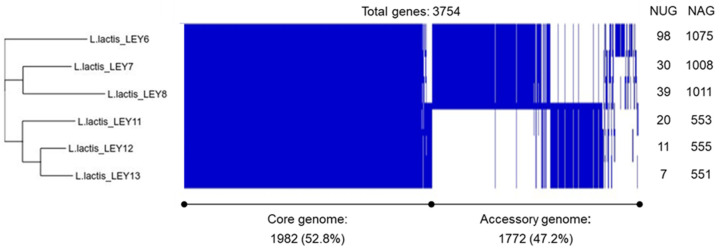
Pangenome analysis of the six GABA-producing *L. lactis* isolates as determined using Roary software. The cluster tree based on 3754 single genes was produced using Phandango software. The number of unique genes (NUG) and accessory genes (NAG) are shown on the right.

Table S1 shows the general genomic information obtained for the six GABA-producing *L. lactis* isolates. Whole-genome assembly revealed draft genome sizes of between 2,513,704 and 2,872,551 bp. After pangenome analysis, a total of 3754 genes were identified. The core genome was composed of 1982 genes. The isolates showed a variable number of unique genes ranging from 7 in LEY13 up to 98 in LEY6. The other 1772 genes were shared between two or more isolates. These results indicate that the six GABA-producing *L. lactis* isolates are different strains. A cluster tree based on the pangenome results was constructed (Figure 2) and revealed the existence of two major groups, one including strains LEY6, LEY7, and LEY8 and the other including LEY11, LEY12, and LEY13. 

### 3.4. In Silico Identification of the GAD Cluster and Other Technologically Relevant Genes in the Genome of the GABA-Producing L. lactis Strains

The GAD gene cluster involved in GABA production was identified in all six strains (Table S2). The genetic organization of this cluster was identical to that previously described in the GABA non-producer *L. lactis* subsp. *cremoris* MG1363 [19]. All six GABA-producing strains had gene clusters involved in the ability to grow in milk, e.g., the lactose phosphotransferase operon (*lacR-lacABCDFEGX*), the genes encoding cell wall-associated proteases (*prtP* and *prtM*), and those coding for the oligopeptide permease system (*oppDFBCA*) (Table S2). They also had putative genes for a citrate/sodium symporter and a malolactic enzyme that might be involved in citrate utilization (Table S2). LEY6, LEY7, and LEY8 had a putative locus for a cluster coding for a bacteriocin similar to macedovicin (annotated as type A2 lantipeptide), while LEY11, LEY12, and LEY13 had another two, one for a bacteriocin similar to carnolysin (annotated as type 2 lantibiotic) and one for another similar to Nisin Z (annotated as a gallidermin/nisin family lantibiotic) (Table S2).

### 3.5. Technological Characterization of the GABA-Producing L. lactis Strains

#### 3.5.1. Acidifying Capacity

The six *L. lactis* strains were able to acidify the milk, with no differences among them, either after 6 h or after 18 h of incubation (*p* < 0.05); all reduced the pH by more than 1 unit (between 1.06 for LEY12 and 1.65 for LEY6) in the first 6 h. A significative reduction in the pH value was found after 18 h for all the strains (*p* < 0.05), from 4.12 ± 0.03 for LEY13 to 4.32 ± 0.31 for LEY7 (Table 3). All the strains completely clotted the milk after 18 h of incubation (Table 3).

#### 3.5.2. Proteolytic Activity

**Figure 3:**
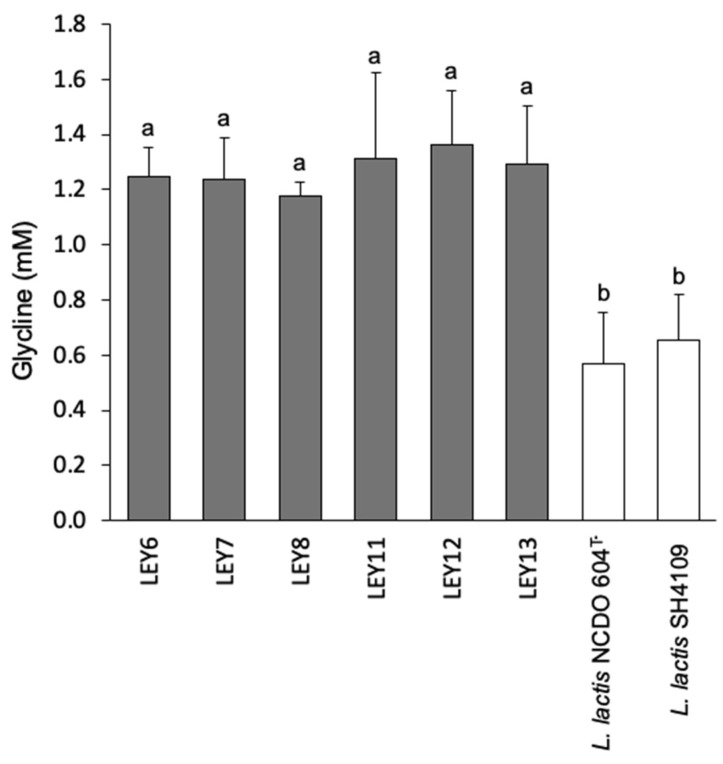
Proteolytic activity of the GABA-producing *L. lactis* strains as determined by the O-phthaldialdehyde (OPA) assay. Proteolytic activity was recorded as millimoles of glycine released after incubation in skimmed cow’s milk at 30 °C for 24 h, using a glycine calibration curve. The strains used as positive control were *L. lactis* NCDO 604^T^ and *L. lactis* SH4109. Bars with different letters indicate significant differences (*p* < 0.05).

All six strains showed a similar proteolytic activity, producing 1.177 ± 0.05 mM Gly for LEY8 to 1.364 ± 0.20 mM Gly for LEY12 (Figure 3). 

In addition, all six strains showed significantly stronger proteolytic activity than *L. lactis* NCDO 604^T^ and *L. lactis* SH4109 (*p* < 0.05) (positive controls). The slight differences observed among the six *L. lactis* strains were not statistically significant.

#### 3.5.3. Dextran Production

None of the *L. lactis* strains produced dextran. 

#### 3.5.4. Production of Volatile Compounds

Table 4 shows the 12 major volatile compounds produce by the six GABA-producing strains. All the strains produced acetaldehyde, 2-methyl propanal, 3-methyl butanal, ethanol, 2-methyl-1-propanol, and 2 or 3-methyl-1-butanol. LEY6 and LEY7 produced the highest amount of 2-methyl-1-propanol and (plus LEY8) of 2 or 3-methyl-1-butanol (*p* < 0.05). No mean differences for the rest of the common compounds were found among the strains. 2-Methyl-butanal was only found, in a similar amount, in LEY6, LEY7, and LEY8 while methyl acetate and acetic acid were found only in LEY11, LEY12, and LEY13. Four strains (LEY7, LEY8, LEY12, and LEY13) produced 2,2,4,6,6 PMH, while only two (LEY11 and LEY12) produced methyl butanoate. Only LEY12 produced acetoin, although in negligible amounts. Strains LEY11 and LEY12 produced the largest number of volatile compounds (10 and 12 respectively).

#### 3.5.5. Production of Antimicrobial Substances

Under the present test conditions, none of the strains produced antimicrobials that inhibited the growth of the indicator strains shown in Table 1.

### 3.6. Safety of GABA-Producing L. lactis Strains

#### 3.6.1. Biogenic Amine Production

None of the six GABA-producing strains produced tyramine, histamine, or cadaverine (Table 5) nor were any corresponding gene clusters found in any of their genomes. However, all the isolates produced putrescine via the agmatine deiminase route (AGDI), but not through the ODC pathway (Table 5). No significant differences were seen among the mean concentrations of putrescine produced by the six strains (*p* < 0.05). 

As expected, the AGDI gene cluster involved in putrescine production in *L. lactis*, which is composed of *aguR* (a positive regulatory gene) followed by the *aguBDAC* operon (encoding the catabolic enzymes for the decarboxylation of agmatine to putrescine and the agmatine/putrescine antiporter gene) [38], was identified in all the strains (Table S2). 

#### 3.6.2. Antimicrobial Resistance Genes

Neither the RGI nor ResFinder analysis detected any antimicrobial resistance genes in any of the six GABA-producing *L. lactis* genomes. 

### 3.7. All Six L. lactis Strains Produced GABA in a Cabrales-Like Mini Cheese Model; None Produced BA

**Figure 4:**
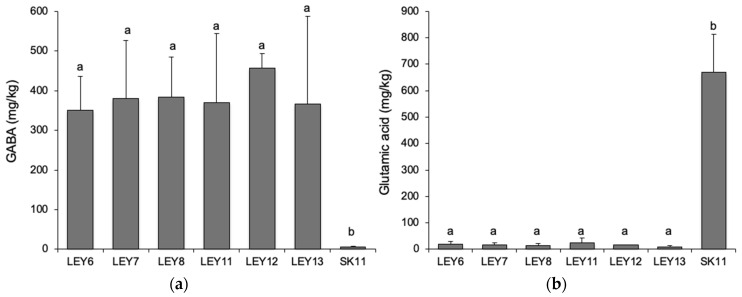
(**a**) GABA and (**b**) glutamic acid accumulation (mg/kg) in Cabrales-like experimental cheeses made using the *L. lactis* strains individually as starter cultures. Bars with different letters indicate significant differences in GABA or glutamic acid accumulation (*p* < 0.05) compared to control cheeses made with the non-GABA producing strain *L. lactis* SK11.

All six tested L. lactis strains were able to accumulate GABA at concentrations ranging from 350 mg/kg (LEY6) to 457 mg/kg (LEY12) (Figure 4a) (no significant difference). 

As expected, significant differences in GABA production were recorded (*p* < 0.05) compared to the control strain *L. lactis* SK11, which produced near-negligible amounts. Apart from the glutamic acid they used in protein biosynthesis, the GABA-producing *L. lactis* strains completely transformed the glutamic acid appearing as a consequence of casein proteolysis into GABA (Figure 4b). In contrast, a mean glutamic acid concentration of about 670 mg/kg remained in the cheeses made with the non-GABA producing *L. lactis* SK11 strain (Figure 4b). None of the cheeses made with any of the six GABA-producing *L. lactis* strains, or *L. lactis* SK11, accumulated any of the BA analyzed, not even putrescine.

## 4. Discussion

GABA, a compound with beneficial effects on human health, is naturally present in many varieties of cheese, although the extent of its accumulation depends on multiple environmental, technological, and metabolic factors [39]. There is much interest in developing functional cheeses with high GABA contents, and the use of GABA-producing *L. lactis* strains with good technological characteristics as starter cultures deserves investigation. In the present work, six *L. lactis* subsp. *lactis* strains that produced GABA in GM17 broth supplemented with monosodium glutamate (Figure 1) were isolated from raw camel’s milk. To our knowledge, this is the first report of the isolation of GABA-producing *L. lactis* strains from milk. Some have been isolated from fermented dairy products such as yogurt [40] and cheese [41,42,43,44], but none has been isolated from the raw milk of any mammal, which has been the dairy source chosen in this work to look for GABA-producing lactococc*i*. The amounts of GABA produced by these *L. lactis* strains ranged from 1.21 mM for LEY13, to 1.80 mM for LEY7, i.e., in the mid-high range reported for these other *L. lactis* strains isolated from other dairy sources than milk [40,42,44].

The technological behavior of the strains agreed with their possession of genomic elements involved in their adaptation to the dairy environment, such as the lactose utilization operon (*lacR-ABCDFEGX*), the proteolytic system (*prtP-prtM*), and the oligopeptide permease system (*oppDFBCA*) (Table S2). Indeed, all six GABA-producing strains showed high acidification rates in milk, the capacity to clot milk (Table 3), and good proteolytic activity (Figure 3). While the acid-producing capacity of a starter is important for milk coagulation, it can also help prevent the growth of spoilage microorganisms and pathogens, and a good proteolytic system contributes to the release of oligopeptides and amino acids during ripening. The catabolism of the volatile compounds produced assists in the development of the final organoleptic characteristics of the fermented product. All six GABA-producing *L. lactis* strains produced different volatile compounds after 24 h of growth in milk (Table 4). Thus, all six showed good technological traits, suggesting they might be of use as functional starter cultures for the production of GABA-enriched fermented dairy products.

*In silico* analysis of the strains’ genomes revealed the absence of any antimicrobial resistance gene or gene cluster involved in the biosynthesis of tyramine, histamine, putrescine through the ODC pathway, or cadaverine. However, all possessed genes responsible for the production of putrescine from agmatine via the AGDI pathway; this is considered a specific trait of *L. lactis*, although many dairy strains have lost it [38]. Although all six strains produced putrescine in culture media supplemented with agmatine (Table 5), neither putrescine nor any other BA was accumulated in any of the experimental cheeses. The absence of agmatine-producing microorganisms in the cheese microbiota would explain why putrescine did not accumulate. In fact, no agmatine production was detected in the control cheeses. However, it cannot be ruled out that GABA-producing *L. lactis* strains would not synthesize putrescine in cheeses that do contain agmatine-producing microorganisms; this needs to be further explored. In this respect, it is worth noting that the elimination of the genes involved in the production of putrescine in *E. faecalis* has no effect on its fitness or the expression of other genes [45]. 

The only discrepancy between the genomic data and the experimental results was the presence of different bacteriocin-producing loci in the genomes of the *L. lactis* strains but a lack of inhibitory activity against the indicator strains tested. However, it should be remembered that *in silico* predictions cannot guarantee that loci are complete and functional. In addition, bacteriocin production in LAB is a highly regulated process involving several factors, and certainly, bacteriocins are produced only under appropriate conditions [46]. Moreover, even though the *L. lactis* strains might have had the capacity to produce bacteriocins, the indicator strains selected might not have been sensitive to them. More work is needed to determine the functionality of the bacteriocin loci identified, the conditions for the production of bacteriocins, and their antibacterial spectrum.

From a technological viewpoint, the most interesting result is that the use of all six GABA-producing *L. lactis* strains as starters led to the accumulation of GABA in the experimental cheeses. The mean GABA concentration of the six cheeses was 384 mg/kg, while the concentration of the cheese made with LEY12 reached 457 mg/kg. There are very few reports on the production of GABA-rich cheeses made with GABA-producing *L. lactis* starter cultures. One describes the use of *L. lactis* subsp. *lactis* biovar diacetylactis 01-7 (approximate inoculum size 2.7 × 10^8^ cfu per mL of pasteurized milk), which is a strain isolated from a cheese starter [17]. However, the mean GABA accumulation was lower (1722 nmol/g [178 mg/kg]) than that achieved in the present work. Another study reports the production of Saint-Paulin experimental cheeses with high concentrations of GABA (from 407–1979 mg/kg) [18] using the same 01-7 strain mentioned above and *L. lactis* subsp. *lactis* biovar diacetylactis 01-1 as starter cultures. GABA accumulation in some of those cheeses was about 4.5 times that of the greatest achieved in the present study (457 mg/kg in cheeses made with LEY12). However, it should be noted that in this earlier work, the cheeses were supplemented with monosodium glutamate (0.3% *w*/*w* fresh cheese), which is a substrate for GABA biosynthesis. Similarly, high concentrations of GABA (up to 766 mg/kg) were reported to accumulate in slurries simulating the composition of Cheddar cheese when supplemented with 3 mg/g of glutamate and inoculated with GABA-producing *L. lactis* subsp. *lactis* ULAAC-H13 and *L. lactis* subsp. *lactis* ULAAC-A23 (isolated from an old-style cheese starter) [47]. The cheeses made in the present study were not similarly supplemented; all the GABA accumulated was synthesized from the glutamate released by casein proteolysis (which was totally consumed and is therefore a limiting factor in GABA production). Taking into account the amount of GABA produced and the tendency of consumers to reject food additives, the six *L. lactis* strains isolated in the present work could be a good technological option. Similar studies by Nomura et al. [18] and Pouliot-Mathieu et al. [12] describe the production of GABA-enriched Cheddar cheeses with no added glutamate but using other *L. lactis* starters. The proteolytic activity of these, added to that of the lactobacilli present in the cheese microbiota, would release this amino acid. In the present work, the GABA-producing *L. lactis* strains were the only starters added, but the Cabrales cheese model used also carries the proteolytic mold *P. roqueforti*, which would have likely caused the appearance of available glutamate.

The parameters used for cheese production in the present work were those routinely used in making Cabrales cheese, but they might be optimized for GABA accumulation. A study by Gardner-Fortier et al. [47] determined the optimal glutamate, pH, and salt-to-moisture ratio conditions for GABA production by *L. lactis* subsp. *lactis* in ripening Cheddar cheese. GABA accumulation increased with the concentration of glutamate and with the lowest salt-to-moisture ratio tested (3%), but the critical factor was a pH of 4.8; at pH 5.1 or pH 5.4, GABA production was very poor. These results might be expected since chloride, glutamate, and low pH are known to regulate the expression of the GAD operon in *L. lactis*, and therefore the biosynthesis of GABA [19,21]. Moreover, the activity of glutamate decarboxylase is enhanced by glutamate and low pH (below 5.0) [18]; the presence of other compounds such as arginine and malate also improve GABA production [48]. Together, these findings suggest that GABA accumulation in cheeses can be increased via the optimization of different technological parameters and the use of starter cultures made by combining the *L. lactis* strains presented in this study with others of technological interest.

GABA-enriched cheeses made using the six investigated strains might be expected to provide health benefits. Inoue et al. [9] showed that a daily intake of 10–12 mg of GABA over 12 weeks via GABA-enriched fermented milk reduced blood pressure in human subjects with mild hypertension. The same was observed when mildly hypertensive men consumed GABA-enriched Cheddar cheese daily (16 mg GABA per day for 12 weeks) [12]. Taking the mean GABA accumulation of 384 mg/kg recorded in the present work, a daily portion of 50 g of cheese (which would provide about 19 mg of GABA) would be enough to have positive effects on human blood pressure; for cheeses accumulating the maximum recorded (457 mg/kg), 23 mg of GABA would be available.

## 5. Conclusions

This work reports the isolation of six GABA-producing *L. lactis* subsp. *lactis* strains from raw camel’s milk collected in Algeria. Genome sequencing revealed all to possess the GAD gene cluster responsible for GABA production, plus genes involved in adaptation to the dairy environment. In addition to showing good technological and safety characteristics, these strains, when used individually as starter cultures, produced experimental Cabrales-like mini cheeses with high GABA concentrations. Therefore, raw Algerian camel’s milk would appear to be an excellent source of GABA-producing *L. lactis* strains with good technological and safety characteristics, which could be used as functional starter cultures for the production of GABA-enriched cheeses beneficial to consumers’ health. 

## Figures and Tables

**Table 1:** Microorganisms used in this study.

Strains/Isolates	Relevant Features	Reference/Source
**Bacterial strains**		
*Latilactobacillus sakei* CECT 906 ^T^	Microbial indicator for bacteriocin production	CECT ^a^
*Lactococcus lactis* subsp. *cremoris* MG1363	Microbial indicator for bacteriocin production	[23]
*Lactococcus lactis* NCDO 604 ^T^	Positive control for proteolytic activity	NCDO ^b^
*Lactococcus lactis* SH4109	Positive control for proteolytic activity	[23]
*Listeria innocua* CECT 910 ^T^	Microbial indicator for bacteriocin production	CECT ^a^
*Micrococcus luteus* NCIMB 8166	Microbial indicator for bacteriocin production	NCIMB ^c^
*Streptococcus thermophilus* CNRZ 1066	Microbial indicator for bacteriocin production	CNRZ ^d^
*Streptococcus thermophilus* LMD9	Microbial indicator for bacteriocin production	NBCC ^e^
*Lactococcus lactis* subsp*. cremoris* SK11	Non-GABA producer starter culture	[24]
**Mold strain**		
*Penicillium roqueforti* 1AM8	Proteolytic mold from Cabrales cheese	[25]
***Lactococcus lactis* isolates**		
*L. lactis* subsp. *lactis* LEY6	GABA producer	This work
*L. lactis* subsp. *lactis* LEY7	GABA producer	This work
*L. lactis* subsp. *lactis* LEY8	GABA producer	This work
*L. lactis* subsp. *lactis* LEY11	GABA producer	This work
*L. lactis* subsp*. lactis* LEY12	GABA producer	This work
*L. lactis* subsp. *lactis* LEY13	GABA producer	This work

^a^ CECT: Spanish Type Culture Collection, Spain. ^b^ NCDO: National Collection of Dairy Organisms, now part of the NCIMB. ^c^ NCIMB: National Collection of Industrial, Food and Marine Bacteria, UK. ^d^ CNRZ: Centre National de Recherches Zootechniques, France. ^e^ NBCC: National Bureau Collection Corporation NCCB, US. ^T^ Type strain.

**Table 2:** Phenotypic characterization of *L. lactis* isolates from Algerian camel’s milk.

*Lactococcus* Milk Isolates	Geographic Location of Milk Samples	Growth at			
		45 °C	pH 9.6	4% NaCl	6.5% NaCl
LEY6	Saida	+	+	+	−
LEY7	Saida	−	+	+	−
LEY8	Saida	−	+	+	−
LEY11	Bechar	+	+	+	−
LEY12	Ghardaia	+	−	+	−
LEY13	Tindouf	+	−	+	−

− Negative; + Positive.

**Table 3:** Acidification kinetics of the *L. lactis* isolates.

*L. lactis* Isolates	Incubation in UHT Skimmed Milk *		
	pH after 6 h	pH after 18 h	Milk Clotting after 18 h
LEY6	5.12 ± 0.26 ^a,a^	4.21 ± 0.13 ^a,b^	+
LEY7	5.33 ± 0.54 ^a,a^	4.32 ± 0.31 ^a,b^	+
LEY8	5.14 ± 0.31 ^a,a^	4.14 ± 0.07 ^a,b^	+
LEY11	5.63 ± 0.33 ^a,a^	4.22 ± 0.12 ^a,b^	+
LEY12	5.71 ± 0.50 ^a,a^	4.18 ± 0.09 ^a,b^	+
LEY13	5.42 ± 0.13 ^a,a^	4.12 ± 0.03 ^a,b^	+

* pH of non-inoculated UHT skimmed milk: 6.77; Values represent the mean ± standard deviation from three independent experiments; the first superscript letter indicates mean comparison among strains for each analyzed time period, while the second superscript letter indicates mean comparison between time periods for each strain; same letter indicates no significant differences (*p* < 0.05); + Total clotting of milk.

**Table 4:** Volatile compounds produced by the *L. lactis* isolates during growth at 30 °C for 24 h in ultra-high temperature (UHT) skimmed milk (HS/GC/MS analysis). Values represent the mean ± the standard deviation of three independent experiments.

	Strains					
Volatile Compound ^†^	LEY6	LEY7	LEY8	LEY11	LEY12	LEY13
Acetaldehyde	76.9 ± 7.5 ^a^	25.7 ± 44.5 ^a^	57.7 ± 52.4 ^a^	23.9 ± 4.3 ^a^	21.9 ± 3.6 ^a^	26.2 ± 8.6 ^a^
2-Methyl propanal	179.1 ± 15.5 ^a^	123.5 ± 43.2 ^a^	180.0 ± 35.3 ^a^	121.3 ± 22.3 ^a^	99.8 ± 30.9 ^a^	131.8 ± 21.9 ^a^
Methyl acetate	− ^b^	^− b^	− ^b^	26.1 ± 7.5 ^a^	25.2 ± 9.4 ^a^	26.9 ± 2.3 ^a^
2-Methyl butanal	72.6 ± 12.9 ^a^	80.1 ± 10.6 ^a^	61.8 ± 16.9 ^a^	− ^b^	− ^b^	− ^b^
3-Methyl butanal	877.7 ± 70.3 ^a^	796.1 ± 197.7 ^a^	797.0 ± 50.3 ^a^	852.8 ± 244.5 ^a^	588.8 ± 248.1 ^a^	897.9 ± 154.6 ^a^
Ethanol	396.5 ± 32.5 ^a^	353.5 ± 107.6 ^a^	340.5 ± 18.8 ^a^	351.5 ± 59.8 ^a^	315.3 ± 32.7 ^a^	362.6 ± 58.5 ^a^
2,2,4,6,6 PMH ^‡^	− ^b^	95.0 ± 164.6 ^a^	66.4 ± 115.1 ^a^	− ^b^	51.1 ± 88.5 ^a^	83.6 ± 144.9 ^a^
Methyl butanoate	− ^b^	− ^b^	− ^b^	2.3 ± 4.0 ^a^	9.1 ± 8.0 ^a^	− ^b^
2-Methyl-1-propanol	55.6 ± 2.2 ^a^	51.4 ± 48.6 ^a^	41.4 ± 7.6 ^b^	30.7 ± 1.8 ^b^	35.7 ± 6.4 ^b^	31.7 ± 7.6 ^b^
Methyl hexanoate	23.9 ± 1.3 ^a^	− ^b^	− ^b^	3.6 ± 6.3 ^a^	2.8 ± 4.8 ^a^	9.6 ± 8.3 ^a^
2 or 3-Methyl-1-butanol	322.9 ± 8.5 ^a^	367.6 ± 28.1 ^a^	298.7 ± 22.8 ^a^	198.4 ± 36.2 ^b^	213.2 ± 42.2 ^b^	205.0 ± 21.5 ^b^
Acetoin	− ^b^	− ^b^	− ^b^	− ^b^	2.6 ± 4.6 ^a^	− ^b^
Acetic acid	− ^b^	− ^b^	− ^b^	21.9 ± 5.7 ^a^	22.1 ± 3.0 ^a^	17.7 ± 2.0 ^a^

^†^ Concentration refers to the internal standard (cyclohexanone 3.6 µg/mL), to which a value of 100 was given; ^‡^ 2,2,4,6,6 Pentamethyl heptane; − Not detected; different superscript letters indicate mean differences among strains, for each compound (*p* < 0.05).

**Table 5:** Biogenic amine production (mM; mean ± standard deviation) in cultures of *L. lactis* isolates.

*L. lactis* Isolates	Tyramine	Histamine	Putrescine (AGDI ^1^)	Putrescine (ODC ^2^)	Cadaverine
LEY6	−	−	0.785 ± 0.02 ^a^	−	−
LEY7	−	−	0.758 ± 0.02 ^a^	−	−
LEY8	−	−	0.763 ± 0.02 ^a^	−	−
LEY11	−	−	0.791 ± 0.02 ^a^	−	−
LEY12	−	−	0.776 ± 0.01 ^a^	−	−
LEY13	−	−	0.803 ± 0.01 ^a^	−	−

^1^ AGDI: agmatine deiminase route; ^2^ ODC: ornithine decarboxylase route; − BA production not detected; ^a^ same superscript letter indicates no significant differences among groups (*p* < 0.05).

## Data Availability

Data is contained within the article or supplementary material. The genome sequences of GABA-producing *L. lactis* strains are openly available in the NCBI BioProject database (BioProject PRJNA596610; http://www.ncbi.nlm.nih.gov/bioproject/596610, accessed on 27 January 2021) under the accession numbers SAMN13634154, SAMN13634584, SAMN13634590, SAMN13634615, SAMN13634616, and SAMN13634617.

## Supplementary Materials

The following are available online at https://www.mdpi.com/2304-8158/10/3/633/s1, Table S1: Genome features of the GABA-producing *L. lactis* isolates; Table S2: Annotation and localization (*locus_tag*) of the main features related to technological, functional and safety properties in the genomes of the GABA-producing *L. lactis* isolates.
