# Supplementary material for: GABA-Producing Lactococcus lactis Strains Isolated from Camel’s Milk as Starters for the Production of GABA-Enriched Cheese
Source: Foods. 2021 Mar 17;10(3):633. doi: 10.3390/foods10030633 (PMC8002479; doi:10.3390/foods10030633)
Supplement: Supplementary file 1 [file foods-10-00633-s001.pdf]

Table S1. Genome features of the GABA-producing *L. lactis* isolates.

***L. lactis* isolates**	**Genome length (bp)**	**GC content (%)**	**Nº of contigs**	**Largest contig (bp)**	**N50 (bp)**	**Protein coding genes**	**tRNAs**	**rRNAs**
LEY6	2,872,551	35.18	118	218,259	148,151	3057	55	7
LEY7	2,814,330	35.17	114	181,253	146,341	2990	55	7
LEY8	2,815,350	35.17	115	219,716	148,151	2993	55	7
LEY11	2,513,704	34.81	38	194,818	220,736	2535	57	7
LEY12	2,514,212	34.80	36	194,818	220,736	2537	57	7
LEY13	2,513,815	34.80	34	670,398	220,736	2533	57	7

Table S2. Annotation and localization (*locus_tag*) of the main features related to technological, functional and safety properties in the genomes of the GABA-producing *L. lactis* isolates.

Features

**GAD gene cluster**		***L. lactis* LEY6**	***L. lactis* LEY7**	***L. lactis* LEY8**	***L. lactis* LEY11**	***L. lactis* LEY12**	***L. lactis* LEY13**
**Gene annotation**	**Gene**	**Locus_tag**	**Locus_tag**	**Locus_tag**	**Locus_tag**	**Locus_tag**	**Locus_tag**
Rgg/GadR/MutR family transcriptional regulator	*gadR*	GQ675_06340	GRR92_07225	GRR91_06335	GRR90_01045	GRR87_01045	GRR88_02000
Glutamate:gamma-aminobutyrate antiporter	*gadC*	GQ675_06335	GRR92_07230	GRR91_06330	GRR90_01050	GRR87_01050	GRR88_01995
Glutamate decarboxylase	*gadB*	GQ675_06330	GRR92_07235	GRR91_06325	GRR90_01055	GRR87_01055	GRR88_01990
**Lactose phosphotranferase operon**		***L. lactis* LEY6**	***L. lactis* LEY7**	***L. lactis* LEY8**	***L. lactis* LEY11**	***L. lactis* LEY12**	***L. lactis* LEY13**
**Gene annotation**	**Gene**	**Locus_tag**	**Locus_tag**	**Locus_tag**	**Locus_tag**	**Locus_tag**	**Locus_tag**
Lactose phosphotransferase system repressor	*lacR*	GQ675_13430	GRR92_13315	GRR91_13265	GRR90_11865	GRR87_11830	GRR88_12085
Galactose-6-phosphate isomerase subunit LacA	*lacA*	GQ675_13435	GRR92_13310	GRR91_13270	GRR90_11860	GRR87_11835	GRR88_12080
Galactose-6-phosphate isomerase subunit LacB	*lacB*	GQ675_13440	GRR92_13305	GRR91_13275	GRR90_11855	GRR87_11840	GRR88_12075
Tagatose-6-phosphate kinase	*lacC*	GQ675_13445	GRR92_13300	GRR91_13280	GRR90_11850	GRR87_11845	GRR88_12070
Tagatose 1,6-bisphosphate aldolase	*lacD*	GQ675_13450	GRR92_13295	GRR91_13285	GRR90_11845	GRR87_11850	GRR88_12065
PTS system, lactose-specific IIA component	*lacF*	GQ675_13455	GRR92_13290	GRR91_13290	GRR90_11840	GRR87_11855	GRR88_12060
PTS system, lactose-specific IIBC component	*lacE*	GQ675_13460	GRR92_13285	GRR91_13295	GRR90_11835	GRR87_11860	GRR88_12055
6-phospho-beta-galactosidase	*lacG*	GQ675_13465	GRR92_13280	GRR91_13300	GRR90_11830	GRR87_11865	GRR88_12050
Aldose 1-epimerase	*lacX*	GQ675_13475	GRR92_13270	GRR91_13310	GRR90_11820	GRR87_11875	GRR88_12040
**PrtP/PrtM proteolytic system**		***L. lactis* LEY6**	***L. lactis* LEY7**	***L. lactis* LEY8**	***L. lactis* LEY11**	***L. lactis* LEY12**	***L. lactis* LEY13**
**Gene annotation**	**Gene**	**Locus_tag**	**Locus_tag**	**Locus_tag**	**Locus_tag**	**Locus_tag**	**Locus_tag**
Proteinase	*prtP*	GQ675_13980	GRR92_13700	GRR91_13740	GRR90_12010	GRR87_12095	GRR88_11930
Peptidyl-prolyl isomerase, maturation protein	*prtM*	GQ675_13975	GRR92_13705	GRR91_13735	GRR90_12005	GRR87_12090	GRR88_11935

Table S2 (continued)

**Oligopeptide permease system**		*L. lactis* LEY6	*L. lactis* LEY7	*L. lactis* LEY8	*L. lactis* LEY11	*L. lactis* LEY12	*L. lactis* LEY13
**Gene annotation**	**Gene**	**Locus_tag**	**Locus_tag**	**Locus_tag**	**Locus_tag**	**Locus_tag**	**Locus_tag**
Oligopeptide transport ATP-binding protein OppD	*oppD*	GQ675_14520	GRR92_14260	GRR91_14230	GRR90_12400	GRR87_12445	GRR88_12450
Oligopeptide transport ATP-binding protein OppF	*oppF*	GQ675_14525	GRR92_14265	GRR91_14235	GRR90_12405	GRR87_12440	GRR88_12445
Oligopeptide transport system permease protein OppB	*oppB*	GQ675_14530	GRR92_14270	GRR91_14240	GRR90_12410	GRR87_12435	GRR88_12440
Oligopeptide transport system permease protein OppC	*oppC*	GQ675_11395	GRR92_14275	GRR91_14245	GRR90_12415	GRR87_12430	GRR88_12435
Oligopeptide binding protein OppA	*oppA*	GQ675_11390	GRR92_14325	GRR91_14270	GRR90_12440	GRR87_12455	GRR88_12455
**Citrate utilization**		*L. lactis* LEY6	*L. lactis* LEY7	*L. lactis* LEY8	*L. lactis* LEY11	*L. lactis* LEY12	*L. lactis* LEY13
**Gene annotation**	**Gene**	**Locus_tag**	**Locus_tag**	**Locus_tag**	**Locus_tag**	**Locus_tag**	**Locus_tag**
Citrate:sodium symporter	-	GQ675_14140	GRR92_13885	GRR91_13915	GRR90_02815	GRR87_02815	GRR88_00230
Oxaloacetate-decarboxylating malate dehydrogenase	*maeA*	GQ675_14145	GRR92_13880	GRR91_13910	GRR90_02820	GRR87_02820	GRR88_00225
**Bacteriocins[a]**		*L. lactis* LEY6	*L. lactis* LEY7	*L. lactis* LEY8	*L. lactis* LEY11	*L. lactis* LEY12	*L. lactis* LEY13
**Gene annotation**	**Gene**	**Locus_tag**	**Locus_tag**	**Locus_tag**	**Locus_tag**	**Locus_tag**	**Locus_tag**
Type A2 lantipeptide		GQ675_10525	GRR92_10240	GRR91_10245			
Gallidermin/nisin family lantibiotic					GRR90_11940	GRR87_09650	GRR88_12205
Type 2 lantibiotic					GRR90_09375	GRR87_09055	GRR88_09380
Type 2 lantibiotic					GRR90_09380	GRR87_09060	GRR88_09385
**AGDI cluster**		*L. lactis* LEY6	*L. lactis* LEY7	*L. lactis* LEY8	*L. lactis* LEY11	*L. lactis* LEY12	*L. lactis* LEY13
**Gene annotation**	**Gene**	**Locus_tag**	**Locus_tag**	**Locus_tag**	**Locus_tag**	**Locus_tag**	**Locus_tag**
Transcriptional regulator of the putrescine production cluster (AguR)	*aguR*	GQ675_08305	GRR92_07575	GRR91_08020	GRR90_06180	GRR87_06180	GRR88_06185
Putrescine carbamoyltransferase (AguB)	*aguB*	GQ675_08310	GRR92_07580	GRR91_08015	GRR90_06175	GRR87_06175	GRR88_06180

Agmatine/putrescine antiporter (AguD)	*aguD*	GQ675_08315	GRR92_07585	GRR91_08010	GRR90_06170	GRR87_06170	GRR88_06175
Agmatine deiminase (AguA)	*aguA*	GQ675_08320	GRR92_07590	GRR91_08005	GRR90_06165	GRR87_06165	GRR88_06170
Carbamate kinase (AguC)	*aguC*	GQ675_08325	GRR92_07595	GRR91_08000	GRR90_06160	GRR87_06160	GRR88_06165

[a] Bagel software was used to predict putative bacteriocin clusters in the genome of *L. lactis* isolates; only the locus_tag corresponding to the genes encoding bacteriocin peptides is indicated.
